# Effects of Plyometric Training on Explosive and Endurance Performance at Sea Level and at High Altitude

**DOI:** 10.3389/fphys.2018.01415

**Published:** 2018-10-09

**Authors:** David Cristóbal Andrade, Ana Rosa Beltrán, Cristian Labarca-Valenzuela, Oscar Manzo-Botarelli, Erwin Trujillo, Patricio Otero-Farias, Cristian Álvarez, Antonio Garcia-Hermoso, Camilo Toledo, Rodrigo Del Rio, Juan Silva-Urra, Rodrigo Ramírez-Campillo

**Affiliations:** ^1^Laboratory of Cardiorespiratory Control, Faculty of Physiological Science, Pontificia Universidad Católica de Chile, Santiago, Chile; ^2^Centro de Investigación en Fisiología del Ejercicio, Facultad de Ciencias, Universidad Mayor, Santiago, Chile; ^3^Departamento de Educación, Facultad de Educación, Universidad de Antofagasta, Antofagasta, Chile; ^4^Departamento Biomédico, Centro Investigación en Fisiología y Medicina de Altura, Universidad de Antofagasta, Antofagasta, Chile; ^5^Department of Physical Activity Sciences, Research Nucleus in Health, Physical Activity and Sport, Quality of Life and Wellness Research Group, Universidad de Los Lagos, Osorno, Chile; ^6^Laboratorio de Ciencias de la Actividad Física, el Deporte y la Salud, Universidad de Santiago de Chile, Santiago, Chile; ^7^Centro de Excelencia en Biomedicina de Magallanes, Universidad de Magallanes, Punta Arenas, Chile; ^8^Centro de Envejecimiento y Regeneración, Pontificia Universidad Católica de Chile, Santiago, Chile

**Keywords:** reactive strength, jump height, hypoxia, endurance performance, explosive performance, stretch-shortening cycle, elastic energy

## Abstract

Plyometric training performed at sea level enhance explosive and endurance performance at sea level. However, its effects on explosive and endurance performance at high altitude had not been studied. Therefore, the aim of this study was to determine the effects of a sea level short-term (i.e., 4-week) plyometric training program on explosive and endurance performance at sea level and at high altitude (i.e., 3,270 m above sea level). Participants were randomly assigned to a control group (*n* = 12) and a plyometric training group (*n* = 11). Neuromuscular (reactive strength index – RSI) and endurance (2-km time-trial; running economy [RE]; maximal oxygen uptake - VO_2_max) measurements were performed at sea level before, at sea level after intervention (SL +4 week), and at high altitude 24-h post SL +4 week. The ANOVA revealed that at SL +4 week the VO_2_max was not significantly changed in any group, although RE, RSI and 2-km time trial were significantly (*p* < 0.05) improved in the plyometric training group. After training, when both groups were exposed to high altitude, participants from the plyometric training group showed a greater RSI (*p* < 0.05) and were able to maintain their 2-km time trial (11.3 ± 0.5 min vs. 10.7 ± 0.6 min) compared to their pre-training sea level performance. In contrast, the control group showed no improvement in RSI, with a worse 2-km time trial performance (10.3 ± 0.8 min vs. 9.02 ± 0.64 min; *p* < 0.05; ES = 0.13). Moreover, after training, both at sea level and at high altitude the plyometric training group demonstrated a greater (*p* < 0.05) RSI and 2-km time trial performance compared to the control group. The oxygen saturation was significantly decreased after acute exposure to high altitude in the two groups (*p* < 0.05). These results confirm the beneficial effects of sea level short-term plyometric training on explosive and endurance performance at sea level. Moreover, current results indicates that plyometric training may also be of value for endurance athletes performing after an acute exposure to high altitude.

## Introduction

Endurance performance depends on several *aerobic* factors (Coyle, 1995), like maximal oxygen uptake (VO_2_max) and running economy (RE) (Shaw et al., 2014), that is the energy expenditure at different velocities (Saunders et al., 2004a). However, endurance performance may also depend on neuromuscular characteristics like reactive strength index (RSI), muscle strength, stiffness, among others (Noakes, 1988; Sinnett et al., 2001). In fact, some *aerobic* endurance determinants like RE (Conley and Krahenbuhl, 1980; Saunders et al., 2004a) can be affected by neuromuscular variables (Turner et al., 2003). More so, neuromuscular performance (i.e., jump-related explosive muscle actions) has been related with endurance performance at different distances (Hudgins et al., 2013). Thus, the energy cost of running reflects the sum of both *aerobic* and *anaerobic* (neuromuscular factors) metabolism (Daniels, 1985). Hence, training strategies that can increase both *aerobic* and neuromuscular factors related with endurance performance would be of great value for endurance athletes.

Plyometric training (i.e., a jump-based strength training method) (Markovic and Mikulic, 2010) is a commonly used training strategy to increase neuromuscular strength by means of stretch-shortening cycle muscle actions (Ramirez-Campillo et al., 2013) and may positively affect *aerobic*-related endurance performance variables (i.e., RE) (Conley and Krahenbuhl, 1980; Saunders et al., 2004a), possible through increasing RSI (Paavolainen et al., 1999). In fact, a previous study reported that after a 6-week period of plyometric training recreational runners improved their RE (Turner et al., 2003) and this increase also can be expected in highly trained middle and long distance runners (Saunders et al., 2006). Moreover, plyometric training can increase RE independently from VO_2_max changes (Paavolainen et al., 1999). This phenomenon is important in highly trained endurance athletes due to their limited ability to increase VO_2_max (Midgley et al., 2007). Furthermore, plyometric training has a positive effect on time trial performance (Paavolainen et al., 1999) and endurance athletes with better performance may achieve better adaptive responses to plyometric training (Ramirez-Campillo et al., 2014a). Therefore, plyometric training may increase RE and RSI, which may positively affect endurance performance (Pollock, 1977; Thomas et al., 1999). However, whether the positive effects of plyometric training on *aerobic* and neuromuscular performance variables are still present after short term exposure at high altitude (HA) remain unexplored.

Acute exposure (<24 h) to high altitude decreases VO_2_max (Maher et al., 1974; Young et al., 1996; Bassett and Howley, 2000) and endurance performance (Maher et al., 1974; Fulco et al., 1998). Specifically, in well trained subjects, small but significant *aerobic* performance impairments may occur even at an altitude of ∼540 m, with reductions in *aerobic* performance up to ∼35% at higher altitudes (Fulco et al., 1998). This phenomenon can be explained by a decrease in partial pressure and arterial saturation in O_2_ with a lower barometric pressure in HA (Wagner, 2010), negatively affecting VO_2_max. Additionally, extrapolation of data showed that VO_2_max fell 0.9% per every 100 m above altitude ≥1,100 m (Vogt and Hoppeler, 2010). However, neuromuscular variables related to fitness performance (i.e., jump) may not be negatively affected by acute exposure to high altitude (Coudert, 1992; Kayser et al., 1993; Burtscher et al., 2006). In fact, neuromuscular performance variables may even be favorably affected (Wood et al., 2006). For example, in the Olympic Games of 1968, held at 2,300 m above sea level, several world records in maximal-intensity and short-durations events were improved, such as the long jump (Berthelot et al., 2015). Moreover, among elite athletes, jumping performance is usually improved above 1,500 m above sea level (Hamlin et al., 2015). In a recent study (Garcia-Ramos et al., 2018), young male and female swimmers (age, ∼19 years) were acutely exposed to 2,320 m above sea level, and their explosive performance during a loaded jump-task was increased when compared to sea-level performance in terms of maximal velocity (up to 7.6%), maximal power (up to 6.8%) and peak force (up to 3.6%). Similar findings were also reported in physically active subjects after performing repeated jumps under high-hypoxic conditions (Alvarez-Herms et al., 2015). As endurance performance depends on both *aerobic* and neuromuscular variables (Daniels, 1985), it is possible that the endurance performance can be benefited per neuromuscular variables on high altitude. Therefore, this study was aimed to evaluate if a short-term (i.e., 4 weeks) sea level plyometric training program could affect explosive and endurance performance on sea level and at high altitude (i.e., 3,270 m), through its positive effects on neuromuscular performance variables (i.e., RSI) and aerobic determinants of endurance performance (i.e., 2 km time trial test) at sea level (Turner et al., 2003), independently from changes in VO_2_max (Paavolainen et al., 1999).

## Materials and Methods

### Participants

A group of participants was submitted to neuromuscular (30 cm drop jump reactive strength index [RSI30]) and endurance (2-km time-trial; running economy; VO_2_max) measurements at sea level before (SL) and after intervention (SL +4 week) and again (RSI30; 2-km time-trial) after 24-h post SL +4 week at high altitude (HA +4 week).

Physically active (i.e., recreational runners) lowlanders participants (sixteen males and seven females; height: 162.6 ± 9.4 cm; body mass: 62.2 ± 12.4 kg; age 21.3 ± 1.3 years) were recruited, and randomly assigned to a control group (*n* = 12, 8 males, and 4 females) and plyometric training group (n = 11, 8 males, and 3 females). Although not matched for any specific dependent variable, all measurements taken at baseline (at sea level and at high altitude) in both the control and the plyometric training group were homogeneous.

None of the participants had any background (in the 6-month period preceding the study) in regular strength training or competitive sports activity that involved any kind of jumping training exercise used during the treatment. Sample size was computed based on the changes observed in the reactive strength index (Δ = 0.33 mm⋅ms^-1^; *SD* = 0.3) after a short-term plyometric training study (Ramirez-Campillo et al., 2014b). Exclusion criteria considered (i) potential medical problems or a history of ankle, knee, or back injury, (ii) any lower extremity reconstructive surgery in the past two years or unresolved musculoskeletal disorders, (iii) history of acute mountain sickness, (iv) previous (≤2 months) pre-acclimation at high altitude exposure (3,000 m above sea level). Additionally, as physical performance at high altitude was a major dependent variable, participants were excluded from the study if they experienced acute mountain sickness during exposure to high altitude.

Institutional review board approval for our study was obtained from the ethical committee of the Universidad de Antofagasta. All participants were carefully informed about the experiment procedures, and about the possible risk and benefits associated with their participation in the study, and an appropriate signed informed consent document has been obtained in accordance with the Declaration of Helsinki. We comply with the human and animal experimentation policy statements guidelines of the American College of Sports Medicine.

### Experimental Design

**FIGURE 1:**
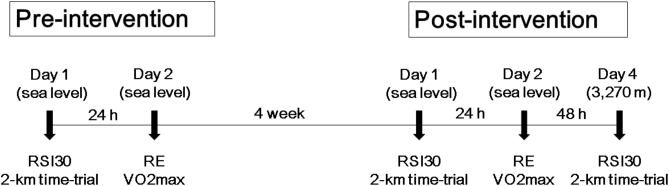
Schematic representation of the study experimental design. RSI30, 30 cm drop jump reactive strength index; RE, running economy; VO_2_max, maximal oxygen uptake.

A schematic representation of the experimental study design is depicted in **Figure 1**.

The participants were carefully familiarized with the tests procedures during several submaximal and maximal exercises before the measurements were taken. The participants also completed several explosive trials to become familiar with the exercises used during training. In addition, several warm-up muscle actions were performed prior to the actual maximal and explosive test actions (Andrade et al., 2015). Tests were always administered in the same order, time of day and by the same investigator, on non-consecutive days before and after (i.e., ≥48 h after last training session) the 4 weeks of intervention. Participants were instructed to (i) have a good night’s sleep (≥8 h) before each testing day, (ii) have a meal rich in carbohydrates and to be well hydrated before measurements, (iii) use the same athletic shoes and clothes during the pre- and post-intervention testing, (iv) give their maximum effort during the performance measurements. On day one height, body mass, RSI30 and 2-km time trial test were measured. On day two, RE and VO_2_max were measured. After the second day of measurements, participants took an autobus during 5 h to ascended at 3,270 m above sea level (Caspana city, Antofagasta Region, Chile), spending 24 h at this elevation before measurements of RSI30 and 2-km time trial tests (HA +4 week). Considering that there are relatively few published studies regarding the acute effects of high altitude on participants that ascent to high altitude after plyometric training (Khodaee et al., 2016), it was deemed of relevance to conduct measurements after an acute exposure of 24 h at 3,270 m above sea level.

Standard warm-up (i.e., 5 min of submaximal running with several displacements, 20 vertical and 10 longitudinal jumps) was executed before each testing day. In addition, height was measured using a wall-mounted stadiometer (HR-200, Tanita, Japan) recorded to the nearest 0.5 cm. Body mass was measured to the nearest 0.1 kg using a digital scale (BF-350, Tanita, Illinois, United States).

Additionally, in order to discard participants suffering acute mountain sickness, at SL +4 week and HA +4 week a self-administered questionnaire from Lake Louise Acute Mountain Sickness Scoring system (Roach, 1993) was applied to participants. A score ≥3 points discarded the participation from the study (Roach, 1993). To verify physiological changes of acute exposure to hypoxia, systolic blood pressure (SBP), diastolic blood pressure (DBP), mean arterial blood pressure (MABP), heart rate (HR), and oxygen saturation (SpO_2_) were measured at SL +4 week and at HA +4 week. According to the Lake Louise acute mountain sickness survey, and physiological recordings, none of the participants suffer acute mountain sickness (**Table 1**). Therefore, all subjects were considered eligible for inclusion in this study.

**Table 1:** Effect of high altitude (3,270 m above sea level) on physiological parameters and Lake Louise acute mountain sickness survey.

	Sea Level		24 h. post high altitude	
	Control *n* = 12	Plyometric *n* = 11	Control *n* = 12	Plyometric *n* = 11
SBP (mmHg)	109.0 ± 11.5	110.1 ± 8.9	127.7 ± 16.1	108.1 ± 10.4
DBP (mmHg)	57.0 ± 8.6	66.6 ± 5.1	62.9 ± 8.7	63.1 ± 7.2
MABP (mmHg)	74.3 ± 8.9	81.1 ± 5.4	84.5 ± 10.0	78.2 ± 7.7
HR (beats/min)	63.8 ± 9.5	63.3 ± 6.8	77.9 ± 17.4	76.3 ± 10.5
SpO_2_ (%)	99.1 ± 1.1	98.2 ± 0.1	89.8 ± 3.2^∗^	93.1 ± 0.1^∗^
Headache	*-*	*-*	4 (33%)	0 (100%)
Gastrointestinal	*-*	*-*	0 (100%)	0 (100%)
Fatigue and/or weakness	*-*	*-*	0 (100%)	1 (27.3%)
Dizziness/lightheadedness	*-*	*-*	0 (100%)	1 (27.3%)
Difficulty of sleeping	*-*	*-*	0 (100%)	2 (45.5%)


*Data are present as mean ±*SD*. SBP, systolic blood pressure; DBP, diastolic blood pressure; MABP, mean arterial blood pressure; HR, heart rate; SpO_*2*_, oxygen saturation. ^∗^*p* < 0.05 vs. Sea level.*

### RSI30 Test

A detailed description of this test is reported elsewhere (Ramirez-Campillo et al., 2013). Briefly, participants performed drop jumps from a 30 cm height platform, using an electronic contact mat system (Globus Tester, Codogne, Italy). The participants were instructed to place their hands on their hip and step off the platform with the leading leg straight to avoid any initial upward propulsion, ensuring a drop height of 30 cm. They were instructed to jump for maximal height and minimal contact time, in order to maximize jump reactive strength. The participants were again instructed to leave the platform with knees and ankles fully extended and to land in a similarly extended position to ensure the validity of the test. Three repetitions were executed, with at least 2 min of rest between them. The best performance trial was used for the subsequent statistical analysis.

### Time Trial Test

This was the only test performed outdoors. At sea level the relative humidity (i.e., between 66 and 68%) and temperature (i.e., between 19 and 21°C) was similar at baseline and after training intervention. Furthermore, at high altitude, the relative humidity was between 21 and 25% and temperature was 19°C (Chilean Meteorological Service). Participants ran 28.6 laps in a 70-m outdoor concrete track for a total of 2 km. This track was the same at sea level and high altitude. Aside from the standard warm-up, before the time-trial test, participants completed two submaximal laps around the track and 4 min later, they had one maximal attempt to complete the test.

### VO_2_max

The VO_2_max was measured during an incremental test on a treadmill (LifeFitness, model 95Te, United States) to volitional exhaustion, as previously described (Saunders et al., 2004b,c). Briefly, the velocity was increased in 0.5 km.h^-1^ every 30 s from 8.0 km.h^-1^ to 12.0 km.h^-1^, then the inclination of the treadmill was increased 0.5% every 30 s until volitional fatigue. Gas exchange was recorded continuously with a portable breath-to-breath gas analyzer (K4b2, Cosmed, Italy). The analyzer was calibrated according to the manufacturer’s instructions prior to each trial run. Pulmonary ventilation (VE), oxygen uptake (VO_2_), expired carbon dioxide (VCO_2_), and respiratory exchange ratio (RER) were averaged over 10 s periods, with the highest 30 s value (i.e., three consecutive 10 s periods) used in the analysis. VO_2_max was determined according to achievement of previously established criteria (Howley et al., 1995): (i) VO_2_ plateau (increase <150 ml⋅min^-1^), (ii) RER >1.1, and (iii) ≥90% of theoretical maximal heart rate (HRmax). The VO_2_max was expressed relative to body mass (ml⋅kg^-1^⋅min^-1^).

### Running Economy

Running economy (RE) was determined by measuring VO_2_ at three different sub-maximal velocities (i.e., 10.0, 11.0, and 12.0 km⋅h^-1^), on a treadmill (LifeFitness, model 95Te, United States), with 0% of inclination. At each sub-maximal velocity, a 4-min-collection period was employed. Running economy was defined as the mean VO_2_ attained during the last minute of each running speed. Four minutes was deemed as an adequate time frame to reach steady state (Saunders et al., 2004b,c; Saunders et al., 2006). The RE was expressed as ml⋅kg^-1^⋅min^-1^ and relative to VO_2_max (%) values obtained before and after the intervention to avoid potential confounding factors due to potential pre-post changes in VO_2_max values.

### Training Program

The plyometric training was completed at sea level during 4 weeks, 3 days per week (i.e., ≥48 h of rest between sessions). Each session lasted ∼25 min. The same standard warm-up described above (i.e., testing procedures) was used prior to the main part of the training session. Participants completed bounce drop jumps drills from 30 cm to 50 cm boxes with the same technique (and instructions) previously described for the RSI30 test, intended to maximize reactive strength. Participants completed a total of 60 foot contacts per session (i.e., three sets of 10 jumps from each box). This volume has been used in previous studies, obtaining significant benefits (Ramirez-Campillo et al., 2014a). The rest period between repetitions and sets was 15 s (Read and Cisar, 2001) and 2 min, respectively. The same researcher was always present during training sessions, motivating participants to give their maximum effort in each jump. Participants were reminded to maintain their usual physical activity and nutritional habits during the experiment.

During the intervention, participants maintained their habitual running training (i.e., 3–4 sessions per week, 30–60 min per session, at 70–80% of maximum heart rate). During the intervention participants completed a total volume load of 720 jumps.

### Statistical Analyses

All values are reported as mean ± standard deviation (SD). Statistical analyses were performed by GraphPad Prism 6.0 (GraphPad software Inc., San Diego, CA, United States). Normality assumption for all data was checked with Shapiro-Wilk test. ANOVA with 2 factors (group × time of measurement) following Holm-Sidak *post hoc* analysis was used to determine the effect of intervention on VO_2_max and RE. For RSI and 2-km time trial, a Kruskal-Wallis test followed by Dunn′s multiple comparison test was used to determine the effect of intervention. In addition, the effect size (ES) was calculated for each comparison. The α level used for all statistics was 0.05.

## Results

### After Training, at Sea Level (SL +4 Week)

**FIGURE 2:**
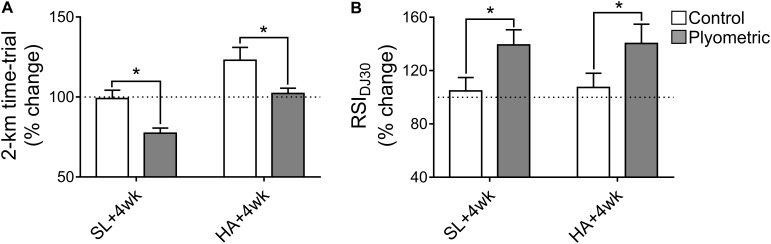
Effect of 4 weeks plyometric training on a 2-km time-trial running test and 30 cm drop jump reactive strength index (RSI_DJ30_). SL +4 week: sea level performance after 4 weeks of plyometric training; HA+4 week: performance after 24 h of high-altitude exposure (3,270-m over sea level) after 4 weeks of plyometric training. **(A)** Note that at SL +4 week the plyometric training group displayed an improvement in 2-km time-trial compared to the control group. At HA +4 week, the control group displayed a reduction in 2-km time-trial performance compared to sea level, and the plyometric training group showed a better performance compared to the control group. **(B)** At SL +4 week and at HA +4 week the plyometric training group displayed an improvement in RSI compared to the control group. ANOVA with 2 factors, followed by Holm-Sidak *post hoc*; ^∗^*p* < 0.05. Dotted line reflects baseline performance at sea-level.

Regarding to the reactive strength index, compared to the sea level pre-training value (taken as 100% of performance), neuromuscular performance was improved after plyometric training (139.3 ± 11.3% of pre-training performance; *p* < 0.05; ES = 0.20), while the control group did not modify its neuromuscular performance (**Figure 2B**). Moreover, when the plyometric training group and the control groups were compared, a greater (*p* < 0.05) reactive strength index was observed in the plyometric training group after training (**Figure 2B**).

In relation to the 2-km time trial performance test, compared to the performance at sea level pre-training (11.3 ± 0.5 min; taken as 100% of performance), after training the plyometric training group improved (reduced) the time needed to complete the test (8.75 ± 0.4 min, 77.4 ± 3.2% of pre-training time; *p* < 0.05; ES = 0.31), while the control group did not modify its performance (**Figure 2B**). Moreover, when the plyometric training group and the control groups were compared, a greater (*p* < 0.05) 2-km time trial performance was observed in the plyometric training group after training (**Figure 2A**).

**FIGURE 3:**
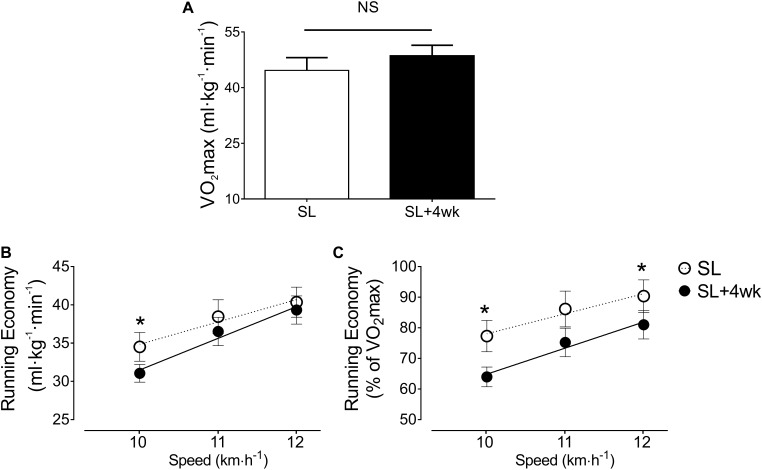
Four weeks of plyometric training improve running economy (RE). **(A)** Sea level maximal oxygen uptake (VO_2_max) (ml⋅kg^-1^⋅min^-1^) before (SL, white column) and after (SL +4 week, black column) 4 weeks of plyometric training. **(B)** Sea level running economy (ml⋅kg^-1^⋅min^-1^) before (SL, white dots) and after 4 weeks of plyometric training (SL +4 week, black dots). Note that at 10 km⋅h^-1^ the plyometric training group showed a decrease of VO_2_ compared to SL pre-training. **(C)** RE normalized to VO_2_ max which showed that at 10 km⋅h^-1^ and 12 km⋅h^-1^ plyometric training group displayed an improvement of RE. ANOVA with 2 factors, followed by Holm-Sidak *post hoc*; ^∗^*p* < 0.05. NS, denotes no significant differences between time periods.

Compared to pre-training VO_2_max values (44.7 ± 3.4 ml⋅kg^-1^⋅min^-1^), the VO_2_max was not significantly affected by plyometric training after 4 weeks (48.6 ± 2.8 ml⋅kg^-1^⋅min^-1^; ES = 0.13) (**Figure 3A**). However, after plyometric training the RE was improved at 10 km⋅h^-1^ (post-training, 31.1 ± 2.8 ml⋅kg^-1^⋅min^-1^; pre-training, 34.5 ± 4.6 ml⋅kg^-1^⋅min^-1^; *p* < 0.05; **Figure 3B**). In addition, the RE normalized to VO_2_max was significantly (*p* < 0.05) improved after training at 10 km⋅h^-1^ (-13.3%; ES = 0.87) and 12 km⋅h^-1^ (-9.4%; ES = 0.76) (**Figure 3C**).

### After Training, at High Altitude (HA +4 Week)

During acute high altitude exposure, SBP, DBP, MABP, and HR were not significant different as compared to sea level (**Table 1**). The SpO_2_ was reduced (*p* < 0.05) in both groups, plyometric (93.1 ± 0.1 vs. 98.2 ± 0.1%, ES = 0.51) and control (89.8 ± 3.2% vs. 99.1 ± 1.1 %, ES = 0.38) after 24 h of high altitude exposure compared to sea level (**Table 1**). Moreover, physiological parameters at sea level and at high altitude were not significant different between plyometric training and control groups (**Table 1**).

Regarding to the reactive strength index, compared to sea level pre-training value (taken as 100% of performance), after training the neuromuscular performance was greater after 24 h of high altitude exposure (140.4 ± 14.5% of pre-training performance; *p* < 0.05; ES = 0.27) in the plyometric training group, while the control group did not modify its neuromuscular performance (**Figure 2B**). Additionally, when the RSI obtained at high altitude was compared to SL +4 week, no significant differences were observed, nor in the plyometric training group nor in the control group (ES = 0.05; **Figure 2B**). Moreover, when the plyometric training group and the control groups were compared, a greater (*p* < 0.05) reactive strength index was observed in the plyometric training group at high altitude (**Figure 2B**).

In the 2-km time trial test, compared to sea level pre-training value (10.7 ± 0.6 min; taken as 100% of performance), the plyometric training group showed no performance deterioration at high altitude (11.3 ± 0.5 min; 102.2 ± 3.3% of pre-training time; *p* > 0.05; ES = 0.03) (**Figure 2A**). On the contrary, the control group suffered a deterioration in the 2-km time trial test at high altitude (10.3 ± 0.8 min; *p* < 0.05; ES = 0.13) compared to performance at sea level before the intervention (9.02 ± 0.64 min) (**Figure 2A**). Moreover, when the plyometric training group and the control groups were compared, a better (*p* < 0.05) 2-km time trial performance was observed in the plyometric training group at high altitude (**Figure 2A**).

## Discussion

The aim of this study was to determine the effects of a sea level short-term plyometric training program on explosive and endurance performance at sea level and after an acute exposure to high altitude (i.e., 3,270 m above sea level). Main findings confirm the beneficial effects of sea level short-term plyometric training on RSI30, 2-km time trial and running economy at sea level, without affecting sea level VO_2_max. Furthermore, main findings expand knowledge about its beneficial effects, reflecting an improved RSI after an acute exposure to high altitude as well as allowing subjects to maintain their pre-intervention sea-level endurance performance when acutely exposed to high altitude. These results are unique, showing that plyometric training, besides optimizing explosive and endurance performance at sea level, may further aid performance at high-altitude.

Current results showed that 4 weeks of plyometric training improves RSI at sea level. Previous studies also observed short-term improvements in RSI after plyometric training (Adams et al., 1992; Ramirez-Campillo et al., 2013; Ramirez-Campillo et al., 2014a,c). As a novelty, current results showed that the adaptations induced by plyometric training on RSI can be fully transferred when subjects are acutely exposed at high altitude. Short-duration maximal-intensity performance does not depend on *aerobic* metabolism (Faiss et al., 2013). The RSI measurement from drop jumps usually takes < 1 s of maximal-effort. In this sense, it was not surprising to observe that RSI performance adaptations were equally expressed at sea level and at high altitude. Moreover, variables related to jumping performance might be maintained during acute exposure to high altitude (Coudert, 1992; Kayser et al., 1993; Burtscher et al., 2006) and may even be favorably affected (Wood et al., 2006). In fact, a previous cross-sectional study (Garcia-Ramos et al., 2018) revealed that an acute ascent to altitude may even induce a significant effect on the force-velocity relationship obtained during a vertical jump task, with greater maximal power and velocity values compared to sea-level. Although our results did not showed an increase in RSI performance after an acute exposure to high altitude, current findings do provide evidence regarding the positive effects of plyometric training conducted at sea level on RSI performance at sea level and at high altitude.

The RE was improved after plyometric training (31.1 ± 2.8 vs. 34.5 ± 4.6 ml⋅kg^-1^⋅min^-1^, SL +4 week vs. SL, respectively, *p* < 0.05; **Figure 3B**), confirming previous findings (Conley and Krahenbuhl, 1980; Saunders et al., 2004a), including results observed among recreational (Turner et al., 2003) and well trained athletes (Saunders et al., 2006). Additionally, current results indicate this improvement was independent from meaningful changes in VO_2_max, also confirming previous findings (Paavolainen et al., 1999). Of note, the improved RE was observed in line with improvements in RSI, suggesting that RSI enhancement may underlie RE improvements (Paavolainen et al., 1999). These findings may be particularly relevant among highly trained endurance athletes, due to their limited ability to improve VO_2_max (Midgley et al., 2007). Moreover, considering that the mechanisms underlying improvements in RE after a short-term plyometric training are of neuromuscular nature (Markovic and Mikulic, 2010; Balsalobre-Fernandez et al., 2016), such neuromuscular variables related to RE mechanisms may not be negatively affected by acute exposure to high altitude (Coudert, 1992; Kayser et al., 1993; Burtscher et al., 2006), and may be even potentiated (Garcia-Ramos et al., 2018), helping toward a better RE at high altitude.

Moreover, 2-km time trial performance improved after plyometric training, in line with previous findings (Paavolainen et al., 1999; Ramirez-Campillo et al., 2014a). As VO_2_max was not improved after intervention, the time-trial performance improvement may rely on RE and neuromuscular-related (i.e., RSI) adaptations (Coyle, 1995). Different to jumping performance, acute exposure (24 h) to high altitude decreases endurance performance (Maher et al., 1974; Young et al., 1996; Bassett and Howley, 2000). In this context, it is of note that subjects from current study were able to maintain their pre-intervention sea-level endurance performance when acutely exposed to high altitude after short-term plyometric training. This result contrasts with the result observed in the control group that, compared to their pre-intervention sea-level endurance performance, suffered a performance reduction when exposed to high altitude. Thus, it is possible that the improvement of RE and RSI after plyometric training may have enhanced time-trial endurance performance at sea level (Pollock, 1977; Thomas et al., 1999), but also it is possible that these physiological adaptations may have attenuated the negative effects of HA on 2-km time trial performance after acute exposure at high altitude.

As practical application, with only 15 min per day, 3 days per week, 4 weeks of plyometric training may improve endurance and neuromuscular performance at sea level, through adaptations that may be transferred to help subjects perform better after acute exposure to high altitude. This may be of critical importance for endurance athletes competing in hypoxic environments, as *aerobic* performance decreases after acute exposure to high altitude (Fulco et al., 1998). In this sense, for athletes whose performance relies heavily on endurance-related variables, such as soccer players, long-distance runners, among others, their training schedules in preparation for high-altitude competitions may consider the inclusion of key plyometric training protocols in order to maximize their performance.

Some potential limitations should be acknowledge. In this sense, our experimental sample size was limited. Moreover, VO_2_ max and RE were not assessed at high altitude, limiting the possibility to better understand the mechanisms behind the improved 2-km time trial performance in the plyometric training group compared to the control group after an acute exposure to high altitude. Although the current study did not incorporate measurements of RE at high-altitude, it is reasonable to assume that RE also improved at high-altitude, given the aforementioned relationship between RE and RSI (Paavolainen et al., 1999), the latter being improved at high-altitude after plyometric training. Our findings are also limited to the acute effects of high altitude, with more research needed to assess the effects of plyometric training performed at sea level on endurance and explosive performance during more prolonged periods of high altitude exposure. These potential limitations should be taken into account in the interpretation of current findings.

In conclusion, 4 weeks of sea level short-term plyometric training improves RSI30, 2-km time trial and running economy at sea level, without affecting sea level VO_2_max. Moreover, after training, both at sea level and at high altitude, the plyometric training group demonstrated a greater RSI and 2-km time trial performance compared to the control group. These results confirm the beneficial effects of sea level short-term plyometric training on explosive and endurance performance at sea level. In addition, current results indicates that plyometric training may also be of value for endurance athletes performing after an acute exposure to high altitude.

## Author Contributions

DCA, AB, JS-U, CA, AG-H, RDR, and RR-C designed the work and contributed to analysis and interpretation of the data. DCA, AB, CL-V, OM-B, ET, PO-F, CT, and JS-U acquired the data. DCA, AB, CL-V, OM-B, ET, PO-F, CT, JS-U, DCA, CA, AG-H, RDR, and RR-C drafted the work. DCA, AB, CL-V, OM-B, ET, PO-F, CT, JS-U, CA, AG-H, RDR, and RR-C critically revised the work, approved the final version to be published, and agreed to be accountable for all aspects of the work in ensuring that questions related to the accuracy or integrity of any part of the work were appropriately investigated and resolved.

## Conflict of Interest Statement

The authors declare that the research was conducted in the absence of any commercial or financial relationships that could be construed as a potential conflict of interest.
